# The Development and Validation of the Osteoporosis Prevention and Awareness Tool (OPAAT) in Malaysia

**DOI:** 10.1371/journal.pone.0124553

**Published:** 2015-05-04

**Authors:** Li Shean Toh, Pauline Siew Mei Lai, David Bin-Chia Wu, Kok Thong Wong, Bee Yean Low, Claire Anderson

**Affiliations:** 1 Faculty of Science, School of Pharmacy, University of Nottingham, Semenyih, Selangor, Malaysia; 2 Department of Primary Care Medicine, University of Malaya Primary Care Research Group (UMPCRG), Faculty of Medicine, University of Malaya, Kuala Lumpur, Wilayah Persekutuan, Malaysia; 3 School of Pharmacy, Monash University Malaysia, Selangor, Malaysia; 4 Division of Social Research in Medicine and Health, School of Pharmacy, University of Nottingham, Nottingham, United Kingdom; Tufts University, UNITED STATES

## Abstract

**Objectives:**

To develop and validate Osteoporosis Prevention and Awareness Tool (OPAAT) in Malaysia.

**Methods:**

The OPAAT was modified from the Malaysian Osteoporosis Knowledge Tool and developed from an exploratory study on patients. Face and content validity was established by an expert panel. The OPAAT consists of 30 items, categorized into three domains. A higher score indicates higher knowledge level. English speaking non-osteoporotic postmenopausal women ≥50 years of age and pharmacists were included in the study.

**Results:**

A total of 203 patients and 31 pharmacists were recruited. Factor analysis extracted three domains. Flesch reading ease was 59.2. The mean±SD accuracy rate was 0.60±0.22 (range: 0.26-0.94). The Cronbach’s α for each domain ranged from 0.286-0.748. All items were highly correlated (Spearman’s rho: 0.761-0.990, p<0.05), with no significant change in the overall test-retest scores, indicating that OPAAT has achieved stable reliability. Pharmacists had higher knowledge score than patients (80.9±8.7vs63.6±17.4, p<0.001), indicating that the OPAAT was able to discriminate between the knowledge levels of pharmacists and patients.

**Conclusion:**

The OPAAT was found to be a valid and reliable instrument for assessing patient’s knowledge about osteoporosis and its prevention in Malaysia. The OPAAT can be used to identify individuals in need of osteoporosis educational intervention.

## Introduction

The validation of an instrument is necessary to ensure that the cultural differences and language used are suitable for a population, and that the instrument measures what it was designed to measure [1,2]. Seven knowledge tools for osteoporosis have been developed and validated: the Facts on Osteoporosis [3,4,5], the Osteoporosis Knowledge Assessment Tool (OKAT) [6], the Osteoporosis Questionnaire (OPQ) [7], the Osteoporosis Knowledge Test (OKT) [8], the Osteoporosis and You [9], the Osteoporosis Knowledge Questionnaire (OKQ) [10], and the Malaysian Osteoporosis Knowledge Tool (MOKT) [11]. All these tools were developed and validated in English, and were conducted in Australia [6], United Kingdom [7], United States [3,4,5,8,10] Canada [9] and Malaysia [11]. These tools focused mainly on assessing knowledge of osteoporosis and its treatment [3,4,5,6,7,8,9,10,11].

Knowledge of osteoporosis plays an important role in developing attitudes towards the disease which in turn impacts health care behaviors [12]. Patients’ health beliefs are defined by attitudes, values and knowledge about health and health services. Although knowledge is not the only component to cause behavioural changes in patients, it is one of the essential components. Therefore patients should be equipped with the knowledge of the various prevention measures available to increase the likelihood of osteoporosis prevention and its fractures. This includes knowledge on physical activity, adequate calcium intake, adequate vitamin D intake, fall prevention and screening of osteoporosis [13].

Primary prevention of osteoporosis is directed at identifying high risk non-osteoporotic individuals, while secondary prevention of osteoporosis refers to the early detection of the disease and prevention of subsequent fragility fracture. Both primary and secondary prevention involve osteoporosis preventing behaviours [14]. Therefore, it is important to educate patients on the importance of screening and prevention, as studies have found that early detection of osteoporosis are the most cost-effective ways to reduce the number of hospital admittance due to osteoporotic fractures [15,16,17,18].

Although there are many methods to increase osteoporosis preventive behaviour such as physician reminders [16] and screening programs [19], patient education has been found to be an effective component in increasing knowledge and frequency of osteoporosis preventive behavior [20,21,22,23]. However, some studies suggest otherwise [24,25]. The differences in these studies’ methodologies make it difficult to generalize results, as some studies used qualitative methods [26] whilst others used quantitative methods [23,24,25]. The variations in the results also suggest that knowledge is not the only component that affects behavioural change. Beliefs, attitudes and values may also be a barrier to implementing osteoporosis preventive efforts [12].

In Malaysia, the MOKT [11] and the Malay version of the OKT [8,27] have been validated. However, we wanted to assess the knowledge of osteoporosis and its prevention. Hence, these tools were unsuitable for use in our study as the MOKT assessed knowledge on osteoporosis and its treatment, while the OKT assessed osteoporosis knowledge by asking participants to rate the likelihood of getting osteoporosis based on the type of preventive measure taken [8,11]. Hence, the aim of our study was to develop and validate the English version of the Osteoporosis Prevention and Awareness Tool (OPAAT) in Malaysia.

## Method

This study was divided into 2 phases: the development of the OPAAT, and its validation.

### Phase 1: The development of the Osteoporosis Prevention and Awareness Tool (OPAAT)

Despite Malay being the national language of Malaysia, postmenopausal women aged 50 years and above are more fluent in English as schooling was only conducted in the English language then. Hence, the OPAAT was developed in English, based on modifications from the MOKT [11] and findings from a qualitative study which examined the barriers and needs towards an osteoporosis screening and prevention service in Malaysia [28].

We took 10 out of the 50 items from the MOKT, as the other items were related to assessing knowledge on risk factors of osteoporosis, osteoporosis medication or misconceptions about osteoporosis. Six items were rephrased. For item 1, we added the word “fracture” in parenthesis to emphasize that the word “broken bones” means fracture (S1 Table). For item 5, “early on” was removed as patients were unaware that osteoporosis was asymptomatic and the phrase “early on” may confuse them [28]. As for item 13 and 16, we combined the original four questions to develop two questions; as “a loss of height” and “hunchback” were essentially assessing the same thing, and “joint pain” and “swelling of the fingers” were both referring to symptoms of osteoarthritis. Four items from the MOKT were used in its original format.

Results from the qualitative study found that patients, nurses, general practitioners, pharmacists and policy makers lacked knowledge in the following areas: screening and prevention of osteoporosis, and misconceptions of osteoporosis [28]. Therefore 22 new items were added. The final OPAAT consists of 30 items, and was divided into three domains: osteoporosis in general (domain A), consequences of untreated osteoporosis (domain B) and osteoporosis prevention (domain C).

Face and content validity of the OPAAT was established via consultation with an expert panel consisting of four pharmacists with many years of research and clinical experience. Comprehension of the questionnaire was tested on 10 postmenopausal women who understood English. This involved asking the patients for their opinions about the phrasing, format and content of the tool. The patients encountered no difficulty in answering the questionnaire. Hence, no further changes were made.

### Phase 2: The validation of the Osteoporosis Prevention and Awareness Tool (OPAAT)

#### Design

This cross-sectional study was conducted at a primary care clinic of a tertiary hospital from October 2013 to January 2014.

#### Participants in the patient group

English speaking postmenopausal women aged 50 years and above, who had not been diagnosed with osteoporosis/osteopenia was included (This information was obtained from the patient’s medical records). Participants who were feeling too unwell to participate in the study were excluded. The OPAAT was administered to the patient group at baseline and 2 weeks later to assess for reliability.

#### Participants in the professional group

To assess discriminative validity, pharmacists were recruited from the same tertiary hospital. Pharmacists were expected to have a higher knowledge of osteoporosis than patients. The OPAAT was administered to the pharmacists only once, as we wanted to assess the instrument’s ability to discriminate between the knowledge scores of patients and healthcare professionals at baseline.

#### Sample size for the patient group

Sample size was calculated based on a 5:1 participant ratio for factor analysis [29]. Since the OPAAT had 30 items, the total number of participants needed was 150. Allowing for a 20% loss to follow up, the final number of participants required was 180.

#### Sample size for the professional group

The total number of pharmacists recruited was based on the number of pharmacists working in the hospital understudy. This group of participants was excluded from factor analysis.

Instruments used- Osteoporosis Prevention and Assessment tool (OPAAT)The OPAAT consist of 30 items with three domains: osteoporosis in general, consequence of untreated osteoporosis and osteoporosis preventive measure. A score of one was given for a correct response and zero for an incorrect or do not know response. The total score was converted into percentage ranging from 0–100. Each domain score was also analyzed.

#### Procedure

Patients were recruited at the waiting area outside the general practitioner’s consultation room as the waiting time to see the general practitioner’s appointment ranges from one to two hours. Utilising this period of waiting allowed the research team to collect data without extending the duration of the patient’s visit to the hospital.

A 1:2 systematic random sampling method was used to recruit participants, as it was not possible for one researcher to recruit all the eligible participants at the clinic. The medical folders of eligible participants were labelled from 1–40, and a number was randomly drawn from a bag to determine the starting number at the start of each day. This was performed to ensure that sampling was random. Subsequently every 2^nd^ medical folder was selected for recruitment.

Additionally, 11 participants were also recruited using the “snowballing” method. As the project went on, participants began to refer their friends and family. Although this was a non-randomized method of recruiting patients, only 11 (7.3%) participants were recruited in this manner.

The study was explained to the participants using an information sheet. Patient’s written consent was obtained. Baseline demographic information such as patients’ medical history, lifestyle and medication history was collected. Patients answered the questionnaire themselves. For those who experienced some difficulty in reading the questions, the researcher assisted them. The researcher then checked the questionnaire to ensure that all questions were answered. This took approximately 10 minutes. The OPAAT was administered again to the same group of patients after two weeks to assess for reliability. A duration of two weeks was selected for retest, as this time interval is generally accepted to be long enough for participants not to have remembered their original responses, and not long enough for their knowledge of the subject to have changed [30]. Patients were questioned if any significant changes or events occurred within the past two weeks, and all changes were documented.

Pharmacists’ baseline information, work experience and education level were also collected using a baseline information form specific for pharmacist. The OPAAT was administered to the pharmacists only once at baseline.

#### Ethics approval

Written consent was obtained from all participants. This study was approved by the Medical Ethics Committee of the hospital (University Malaya Medical Centre) under study (ref no 920.27).

#### Data analysis

All data was entered into the IBM SPSS version 20 (IBM Corporation, Armonk, NY, USA). Flesch reading ease was calculated using Microsoft Office Word 2007 (Microsoft Corporation, Redmond, WA, USA). Non-parametric tests were used since data obtained were not normally distributed. A p-value <0.05 was considered as statistically significant.

#### Factor analysis

The construct validity of OPAAT was examined using exploratory factor analysis (EFA). Traditionally, factor analysis such as EFA and confirmatory factor analysis (CFA) can only be performed when data are of a continuous scale [31,32]. However, Bruin (2006) developed a new algorithm of EFA to account for categorical data. In this study, EFA was performed on three separate domains to explore the appropriateness of factor structure [33]. Factors with eigenvalues greater than one were considered as having significant contribution in explaining the overall model variation and were retained [34,35].

#### Flesch reading ease

Flesch reading index is a tool used for estimating the reading comprehension level necessary to understand a written document based on the average number of syllables per word and the average number of words per sentence. The Flesch reading ease was calculated using the formula below: Flesch reading ease = 206.835- (1.015x average sentence length)—(84.6 x average number of syllables per word)

The Flesch reading score (which range from 0 to 100) indicates the level of difficulty in understanding the document. The lower the score, the greater the difficulty. An average document should have a score of 60–70 [36].

#### Accuracy rate

The accuracy rate is used to measure the difficulty of a question. It was calculated by the number of correct responses divided by the total number of responses. The higher the accuracy rate, the easier the question was. The optimal level should be 0.5 as a value of higher than 0.75 is deemed to be poor as the question may be too easy. Items with difficulty values between 0.3 and 0.7 are most effective. [37].

#### Cronbach’s α

Cronbach’s α coefficient is a tool used to assess internal consistency. Cronbach’s α value: >0.9- Excellent, >0.8- Good, >0.70- Acceptable, >0.6- Questionable, >0.5- Poor and <0.5- Unacceptable [38]. If omitting an item increases Cronbach’s α significantly, then excluding the item will increase the homogeneity of the scale [39].

Corrected inter-item correlations are the correlations between each item and the total score from the questionnaire. All items should correlate with the total to be considered a reliable scale. A value of less than 0.3 shows a poor correlation and these items should be considered to be excluded. [40].

#### Test-retest for reliability

For test- retest, categorical data were analysed using the kappa measure of agreement and the Mc Nemar’s test. In order to define inter-rater reliability, a kappa measure of agreement was calculated for each item. A kappa value of 0.5 represents moderate agreement, above 0.7 represents good agreement and above 0.8 represents very good agreement [41]. Mc Nemar’s test was used to examine the test-retest reliability on the individual items. Continuous data of the individual items and total domain scores were analyzed using the Wilcoxon signed-rank test and Spearman’s rho correlation coefficient. According to Cohen 1988, a value of 0.10–0.29 showed a low correlation, 0.30–0.49 moderate correlation and 0.50–1.00 high correlation [42].

#### Discriminative validity

To assess discriminative validity, the chi square test was used on categorical data of the individual items to detect the difference between the patient group and professional group. The Mann-Whitney U test was used for continuous data of the individual items and total domains score to compare if there was any significant difference between the patient and professional group.

#### Factors associated with knowledge

Linear multiple regression was used to identify factors associated with knowledge. It used to estimate the linear relationship between a dependent variable (knowledge score) and one or more independent variables (demographic variables).

## Results

A total of 253 patients were approached, 19 declined. 234 participants were recruited (patients = 203, hospital pharmacists = 31), [patient response rate = 91.4%, pharmacists response rate = 100.0%]. Patients’ demographic data are shown in Table 1. Pharmacists recruited worked in different areas of the pharmacy, with working experience ranging from 1–10 years.

**Table 1:** Baseline demographic characteristics of patients.

Characteristics	Patients (n = 203)
**Mean age ± S. D. (years) [range] (Median)**	62.1±7.2 [50–79] (61.0)
**Age range (years) [n (%)]**	
<65	120 (59.1)
≥ 65	83 (40.9)
**Ethnicity [n (%)]**	
Malay	30 (14.8)
Chinese	126 (62.1)
Indian	44 (21.7)
Eurasian	3 (1.5)
**Mean BMI (kg/m** ^**2**^ **) ± S.D. (Median)**	24.2±4.6 (23.3)
**BM I (kg/m** ^**2**^ **) [n (%)]**	
<18.5 (underweight)	10 (4.9)
18.5–24.9 (normal)	118 (58.1)
25.0–29.9 (overweight)	55 (27.1)
≥30.0 (obese)	20 (9.9)
**Level of education [n (%)]**	
Primary (6 years of education)	10 (4.9)
Secondary (11–13 years of education)	78 (38.4)
Diploma/Technical school training (12–14 years of education)	39 (19.2)
Tertiary/Postgraduate (15–21 years of education)	76 (37.4)
**Income per month [n (%)]**	
<RM1000 (<$ 310.7)	36 (17.7)
RM1000-1999 ($ 310.7–621.0)	25 (12.3)
RM2000-2999 ($ 621.3–931.7)	23 (11.3)
RM3000-3999 ($ 932.0–1242.3)	21 (10.3)
RM4000-4999 ($ 1242.6–1553)	17 (8.4)
>RM5000 (>$1553.3)	81 (39.9)

S.D. = standard deviation; BMI = body mass index; $ = US dollar

### Factor analysis

As shown in Table 2, for domain A, EFA yielded one factor with an eigenvalue of 4.04 which contributed to 81.0% of total variation. Ten items within this domain have factor loadings greater than 0.3 in Table 3, suggesting substantial contribution in explaining the overall variation. In Table 4, for domain B, EFA also produced only one factor with an eigenvalue greater of 1.9, which explained 87.3% of the total variation. All five questions within this domain had factor loadings greater than 0.3 as shown in Table 5. In Table 6, for domain C, EFA generated only one factor with an eigenvalue greater than one (4.4). This factor contributed to 69.4% of total variation. Table 7 showed that the factor loadings of all 12 items within this domain were above 0.3. Overall, the data from the three EFAs suggested the adequacy of one factor for each domain (Tables 2–7).

**Table 2:** Eigenvalues of the domain A in the Osteoporosis Prevention and Awareness Tool (OPAAT) using exploratory factor analysis (EFA).

Domain A	Eigenvalue
Factor1	4.04065
Factor2	0.80586
Factor3	0.50583
Factor4	0.22203
Factor5	0.11458
Factor6	0.01873
Factor7	-0.02871
Factor8	-0.10657
Factor9	-0.16125
Factor10	-0.19727
Factor11	-0.22522

**Table 3:** Factor loadings of the domain A in the Osteoporosis Prevention and Awareness Tool (OPAAT) using exploratory factor analysis (EFA).

Variable	Factor1	Factor2	Factor3	Factor4	Factor5	Factor6	Factor7	Factor8
ITEM1	0.3207	0.2394	-0.1778	0.1858	0.2448	0.2203	-0.0682	0.0334
ITEM2	0.3641	-0.2981	0.389	0.1759	0.1214	-0.05	-0.177	-0.0281
ITEM3	0.6867	0.4137	-0.0234	-0.2121	-0.1167	0.0259	-0.1924	0.0187
ITEM4	0.5165	-0.277	0.1993	0.0702	-0.2318	0.104	-0.0083	0.0588
ITEM5	0.7448	0.2325	0.057	0.3106	-0.1576	-0.1444	-0.0722	-0.0153
ITEM6	0.4156	0.4079	0.0697	0.27	0.0308	-0.0044	0.2128	-0.012
ITEM7	0.6944	-0.1801	0.1375	-0.04	0.1071	0.2178	0.0844	-0.0266
ITEM8	0.3345	0.1019	0.359	-0.1986	0.0684	0.0893	0.1261	0.019
ITEM9	0.6472	0.0113	-0.1588	-0.1892	-0.1781	0.172	-0.0105	-0.0556
ITEM10	0.6949	-0.3275	-0.3495	0.0851	0.1654	0.0115	-0.04	0.0059
ITEM11	0.7208	0.0986	0.0598	-0.2682	0.2446	-0.256	0.0009	0.0059
ITEM12	0.8021	-0.264	-0.1945	-0.0087	-0.1141	-0.1628	0.1512	0.0155

Only the factor loadings (represented as eigenvalue) greater than 1 were selected (Harman, 1976)

**Table 4:** Eigenvalues of the domain B in the Osteoporosis Prevention and Awareness Tool (OPAAT) using exploratory factor analysis (EFA).

Domain B	Eigenvalue
Factor1	1.8924
Factor2	0.74467
Factor3	-0.04495
Factor4	-0.19105
Factor5	-0.23417

**Table 5:** Factor loadings of the domain B in the Osteoporosis Prevention and Awareness Tool (OPAAT) using exploratory factor analysis (EFA).

Variable	Factor1	Factor2	Factor3	Factor4	Factor5	Factor6	Factor7	Factor8
ITEM12	0.3207	0.2394	-0.1778	0.1858	0.2448	0.2203	-0.0682	0.0334
ITEM13	0.3641	-0.2981	0.389	0.1759	0.1214	-0.05	-0.177	-0.0281
ITEM14	0.6867	0.4137	-0.0234	-0.2121	-0.1167	0.0259	-0.1924	0.0187
ITEM15	0.5165	-0.277	0.1993	0.0702	-0.2318	0.104	-0.0083	0.0588
ITEM16	0.7448	0.2325	0.057	0.3106	-0.1576	-0.1444	-0.0722	-0.0153

Only the factor loadings (represented as eigenvalue) greater than 1 were selected (Harman, 1976)

**Table 6:** Eigenvalues of the domain C in the Osteoporosis Prevention and Awareness Tool (OPAAT) using exploratory factor analysis (EFA).

Domain C	Eigenvalue
Factor1	4.36008
Factor2	0.84406
Factor3	0.56791
Factor4	0.44087
Factor5	0.31589
Factor6	0.26055
Factor7	0.17115
Factor8	0.01055
Factor9	-0.04459
Factor10	-0.15964
Factor11	-0.21151
Factor12	-0.27104

Only the factor loadings (represented as eigenvalue) greater than 1 were selected (Harman, 1976)

**Table 7:** Factor loadings of the domain C in the Osteoporosis Prevention and Awareness Tool (OPAAT) using exploratory factor analysis (EFA).

Variable	Factor1	Factor2	Factor3	Factor4	Factor5	Factor6	Factor7	Factor8
ITEM17	0.3207	0.2394	-0.1778	0.1858	0.2448	0.2203	-0.0682	0.0334
ITEM19	0.3641	-0.2981	0.389	0.1759	0.1214	-0.05	-0.177	-0.0281
ITEM20	0.6867	0.4137	-0.0234	-0.2121	-0.1167	0.0259	-0.1924	0.0187
ITEM21	0.5165	-0.277	0.1993	0.0702	-0.2318	0.104	-0.0083	0.0588
ITEM22	0.7448	0.2325	0.057	0.3106	-0.1576	-0.1444	-0.0722	-0.0153
ITEM23	0.4156	0.4079	0.0697	0.27	0.0308	-0.0044	0.2128	-0.012
ITEM24	0.6944	-0.1801	0.1375	-0.04	0.1071	0.2178	0.0844	-0.0266
ITEM25	0.3345	0.1019	0.359	-0.1986	0.0684	0.0893	0.1261	0.019
ITEM26	0.6472	0.0113	-0.1588	-0.1892	-0.1781	0.172	-0.0105	-0.0556
ITEM27	0.6949	-0.3275	-0.3495	0.0851	0.1654	0.0115	-0.04	0.0059
ITEM29	0.7208	0.0986	0.0598	-0.2682	0.2446	-0.256	0.0009	0.0059
ITEM30	0.8021	-0.264	-0.1945	-0.0087	-0.1141	-0.1628	0.1512	0.0155

Only the factor loadings (represented as eigenvalue) greater than 1 were selected (Harman, 1976)

### Psychometric properties

Flesh reading ease was 59.2. The mean ± SD accuracy rate was 0.60±0.22 (range: 0.26–0.94). Four out of 30(13.3%) items had values <0.3 and 11/30(36.7%) items had values of >0.75. The remaining 15/30(50.0%) items had values between 0.3–0.75.

Cronbach’s α was analyzed for the three domains. All domains had a Cronbach’s α of ≥0.6 except for domain B (0.286). Thirteen out of 30 items had corrected item –total correlations <0.3 (Table 8).

**Table 8:** Psychometric properties of the Osteoporosis Prevention And Awareness Tool (OPAAT).

Domains	Item Number		Accuracy rate	Cronbach’s α	Corrected Item correlation	Cronbach’s α if item deleted
	1	Makes bones weaker, more brittle and more likely to break (fracture)	0.91		0.421	0.639
	2	Everybody will get osteoporosis as it is part of aging	0.32		0.173	0.672
	3	Osteoporosis occurs because bone is removed faster than it is formed	0.52		0.176	0.673
Osteoporosis in general (A)	4	Osteoporosis and osteoarthritis are different names we can use to describe the same disease	0.58	0.668	0.455	0.619
	5	Osteoporosis usually has no symptoms	0.48		0.065	0.693
	6	Postmenopausal women are not at risk for osteoporosis	0.72		0.416	0.629
	7	Osteoporosis is an untreatable disease.	0.56		0.232	0.663
	8	A bone mineral density test is used to diagnose osteoporosis	0.76		0.428	0.628
	9	I do not need a bone mineral density test unless I fracture my bones.	0.79		0.555	0.608
	10	A bone mineral density test is high in radiation	0.45		0.321	0.646
	11	A bone mineral density test should be performed monthly to monitor bone loss	0.60		0.407	0.629
	12	Results in back pain	0.72		0.272	0.095
	13	Loss of height or hunchback	0.88		0.235	0.173
Consequences of untreated osteoporosis (B	14	Loss of mobility (unable to move around myself)	0.78	0.286	0.164	0.215
	15	Results in tooth loss	0.26		0.006	0.373
	16	Results in joint pain or swelling of fingers	0.27		0.056	0.319
	17	The recommended daily intake for calcium in women above 50 years of age is 1000mg	0.61		0.274	0.744
	18	It is too late to increase calcium intake after the age 50	0.55		0.417	0.727
	19	Glucosamine can help prevent osteoporosis	0.29		0.181	0.753
	20	Calcium supplements can help prevent osteoporosis	0.85		0.397	0.731
	21	The regular dose of calcium supplements can cause kidney stones.	0.26		0.264	0.744
	22	Foods such as milk, tofu, anchovies (*ikan bilis*), yellow dhal and spinach are rich in calcium	0.90	0.748	0.398	0.73
Osteoporosis prevention (C)	23	You can obtain your recommended daily intake of vitamin D via exposing your skin to sunlight for about 15 minutes a day	0.87		0.300	0.739
	24	Increasing coffee and tea intake can help in osteoporosis prevention	0.67		0.479	0.719
	25	Weight bearing exercise (such as brisk walking and line dancing) can decrease bone loss.	0.68		0.248	0.747
	26	Exercise will wear out bones	0.78		0.459	0.723
	27	Certain medications (such as sleeping tablets or high blood pressure medications) may reduce the risk of falling	0.57		0.421	0.726
	28	To prevent falls, comfortable shoes with a good grip should be used.	0.94		0.524	0.728
	29	Poor vision may lead to falls	0.92		0.380	0.734
	30	Being under weight helps prevent osteoporosis	0.60		0.490	0.718
Total Cronbach’s α				0.820		

### Test-retest reliability

At retest, 9(4.4%) patients could not be contacted. Hence, only 194 participants were included at retest (response rate = 95.6%) (See table 9). The Kappa measurement of agreement for 29/30 items (96.7%) were ≥0.8, and 1/30 items (3.3%) was ≥0.7. The McNemar’s test showed no significant differences for all 30 items at test retest. The Wilcoxon signed-rank test showed no significant difference for all domain scores except for the domain on the ‘consequences of untreated osteoporosis.’ However, the total score showed no significant difference. All domains and items were significantly correlated using the Spearman’s rho correlation coefficient (0.760–0.990, p<0.05) (Table 9)

**Table 9:** Test and retest reliability of the individual items for the Osteoporosis Prevention And Awareness Tool (OPAAT).

Domain	Item number	Test (n = 203)			Retest (n = 194)			McNemar’s test p-value	Kappa measurement of agreement* P-value	Spearman’s rho correlation coefficient	Wilcoxon signed-rank test		
		Mean±SD	Median	No. of correct responses [n (%)]	Mean±SD	Median	No. of correct responses [n (%)]				Mean/rank	z-value	P-value
	1	0.91±0.28	1.00	185 (91.1)	0.89±0.32	1.00	172 (88.7)	0.219	0.833	0.838			
	2	0.32±0.47	0.00	64 (31.5)	0.30±0.46	0.00	58 (29.9)	0.250	0.964	0.964			
	3	0.52±0.50	1.00	105(51.7)	0.52±0.50	1.00	101 (52.1)	1.000	0.979	0.979			
Osteoporosis in general (A)	4	0.58±0.50	1.00	117 (57.6)	0.57±0.50	1.00	110 (56.7)	1.000	0.958	0.958			
	5	0.48±0.50	0.00	97 (47.8)	0.48±0.50	0.00	94 (48.5)	1.000	0.990	0.990			
	6	0.72±0.45	1.00	147 (72.4)	0.71±0.46	1.00	137 70.6)	0.508	0.886	0.887			
	7	0.56±0.50	1.00	113 (55.7)	0.54±0.50	1.00	105 (54.1)	0.453	0.927	0.928			
	8	0.76±0.43	1.00	155 (76.4)	0.74±0.44	1.00	144 (74.2)	0.219	0.917	0.918			
	9	0.79±0.41	1.00	160 (78.8)	0.78±0.42	1.00	152 (78.4)	1.000	0.970	0.970			
	10	0.45±0.50	0.00	92 (45.3)	0.46±0.50	0.00	90 (46.4)	0.219	0.938	0.938			
	11	0.60±0.49	1.00	121 (59.6)	0.60±0.49	1.00	118 (60.8)	0.754	0.892	0.893			
	Domain score (%)	60.7±22.2	63.64		60.0±23.8	63.63				0.953	14.54/11.33	-0.724	0.469
	12	0.72±0.45	1.00	147 (72.4)	0.72±0.45	1.00	140 (72.2)	1.000	0.923	0.923			
Consequences of untreated osteoporosis (B)	13	0.88±0.33	1.00	178 (87.7)	0.89±0.31	1.00	173 (89.2)	0.250	0.925	0.927			
	14	0.78±0.42	1.00	158 (77.8)	0.78±0.41	1.00	152 (78.4)	0.500	0.970	0.971			
	15	0.26±0.44	0.00	52 (25.6)	0.27±0.45	0.00	53 (27.3)	0.453	0.908	0.908			
	16	0.27±0.44	0.00	54 (26.6)	0.29±0.45	0.00	56 (28.9)	0.219	0.923	0.924			
	Domain score (%)	58.0±21.3	60.00		59.2±21.7	60.00				0.909	7.50/10.27	-2.216	0.027*
	17	0.61±0.49	1.00	123 (60.6)	0.60±0.49	1.00	116 (59.8)	0.687	0.935	0.936			
	18	0.55±0.50	1.00	112 (55.2)	0.55±0.50	1.00	106 (54.6)	1.000	0.948	0.948			
	19	0.29±0.46	0.00	59 (29.1)	0.28±0.45	0.00	55 (28.4)	1.000	0.962	0.962			
	20	0.85±0.36	1.00	173 (85.2)	0.83±0.38	1.00	161 (83.0)	0.250	0.943	0.945			
Prevention of osteoporosis (C)	21	0.26±0.44	0.00	52 (25.6)	0.26±0.44	0.00	51 (26.3)	0.375	0.932	0.933			
	22	0.90±0.30	1.00	183 (90.1)	0.88±0.32	1.00	171 (88.1)	0.375	0.869	0.872			
	23	0.87±0.34	1.00	176 (86.7)	0.85±0.36	1.00	165 (85.1)	0.453	0.852	0.854			
	24	0.67±0.47	1.00	137 (67.5)	0.68±0.47	1.00	131 (67.5)	0.727	0.905	0.905			
	25	0.68±0.47	1.00	138 (68.0)	0.65±0.48	1.00	126 (64.9)	0.070	0.908	0.910			
	26	0.78±0.41	1.00	159 (78.3)	0.76±0.43	1.00	148 (76.3)	0.289	0.882	0.884			
	27	0.57±0.50	1.00	116 (57.1)	0.55±0.50	1.00	106 (54.6)	0.405	0.760	0.761			
	28	0.94±0.24	1.00	191 (94.1)	0.92±0.28	1.00	178 (91.8)	0.125	0.846	0.856			
	29	0.92±0.28	1.00	186 (91.6)	0.90±0.30	1.00	174 (89.7)	0.250	0.910	0.914			
	30	0.60±0.49	1.00	122 (60.1)	0.59±0.49	1.00	115 (59.3)	1.000	0.947	0.947			
	Domain score (%)	67.8±20.2	71.42		66.4±22.6	71.43				0.937	21.17/19.50	-1.339	0.171
Total OPAAT score (%)		63.6±17.4	66.67		62.9±19.1	66.67				0.950	28.98/27.05	-0.107	0.914

**Statistically significant at p<0.05. Wilcoxon signed-rank test and Spearman’s rho correlation coefficient was used for continuous variables. McNemar’s test and Kappa measurement of agreement was conducted for categorical variables

The overall total knowledge score for the pharmacist group was significantly higher than the patient group (80.9±8.7 vs 63.6±17.4, p<0.001) (Table 10). No significant difference was seen for 16/30(53.3%) items.

**Table 10:** Knowledge scores of the patient and pharmacist group at test.

Domain	Item Number	Patients(n = 203)			Pharmacist(n = 31)			Mann-Whitney U-test			p-value ^a^
		Mean±SD	Median	Participants that answered correctly [n (%)]	Mean±SD	Median	Participants that answered correctly [n (%)]	Mean/rank	Z-value	p-value	
	1	0.91±0.28	1.00	185 (91.1)	0.97±1.80	1.00	30 (96.8)				0.482 ^b^
	2	0.32±0.47	0.00	64 (31.5)	0.58±0.50	1.00	18 (58.1)				0.007*
	3	0.52±0.50	1.00	105(51.7)	0.90±0.30	1.00	28 (90.3)				0.000*
	4	0.58±0.50	1.00	117 (57.6)	0.94±0.25	1.00	29 (93.5)				0.000*
Osteoporosis in general (A)	5	0.48±0.50	0.00	97 (47.8)	0.55±0.51	1.00	17 (54.8)				0.590
	6	0.72±0.45	1.00	147 (72.4)	1.00±0.00	1.00	31 (100.0)				0.002*
	7	0.56±0.50	1.00	113 (55.7)	0.68±0.48	1.00	21 (67.7)				0.284
	8	0.76±0.43	1.00	155 (76.4)	0.94±0.25	1.00	29 (93.5)				0.052
	9	0.79±0.41	1.00	160 (78.8)	0.97±0.18	1.00	30 (96.8)				0.033*
	10	0.45±0.50	0.00	92 (45.3)	0.48±0.51	0.00	15 (48.4)				0.900
	11	0.60±0.49	1.00	121 (59.6)	0.77±0.43	1.00	24 (77.4)				0.088
	Domain score (%)	60.7±22.2	63.64		79.8±12.6	81.82		109.23/171.68	-4.834	0.000*	
	12	0.72±0.45	1.00	147 (72.4)	0.77±0.43	1.00	24 (77.4)				0.713
	13	0.88±0.33	1.00	178 (87.7)	0.84±0.37	1.00	26 (83.9)				0.565 ^b^
Consequences of untreated osteoporosis (B)	14	0.78±0.42	1.00	158 (77.8)	0.81±0.40	1.00	25 (80.6)				0.905
	15	0.26±0.44	0.00	52 (25.6)	0.51±0.51	1.00	16 (51.6)				0.006*
	16	0.27±0.44	0.00	54 (26.6)	0.74±0.44	1.00	23 (74.2)				0.000*
	Domain score (%)	58.0±21.3	60.00		73.6±17.4	80.00		110.98/160.21	-4.086	0.000*	
	17	0.61±0.49	1.00	123 (60.6)	0.58±0.50	1.00	18 (58.1)				0.944
	18	0.55±0.50	1.00	112 (55.2)	0.84±0.37	1.00	26 (83.9)				0.005*
	19	0.29±0.46	0.00	59 (29.1)	0.78±0.43	1.00	24 (77.4)				0.000*
	20	0.85±0.36	1.00	173 (85.2)	0.94±0.25	1.00	29 (93.5)				0.271 ^b^
	21	0.26±0.44	0.00	52 (25.6)	0.61±0.50	1.00	19 (61.3)				0.000*
Prevention of osteoporosis (C)	22	0.90±0.30	1.00	183 (90.1)	1.00±0.00	1.00	31 (100.0)				0.084 ^b^
	23	0.87±0.34	1.00	176 (86.7)	0.81±0.40	1.00	25 (80.6)				0.405 ^b^
	24	0.67±0.47	1.00	137 (67.5)	0.94±0.25	1.00	29 (93.5)				0.006*
	25	0.68±0.47	1.00	138 (68.0)	0.71±0.46	1.00	22 (71.0)				0.900
	26	0.78±0.41	1.00	159 (78.3)	0.84±0.37	1.00	26 (83.9)				0.638
	27	0.57±0.50	1.00	116 (57.1)	0.94±0.25	1.00	29 (93.5)				0.000*
	28	0.94±0.24	1.00	191 (94.1)	0.97±0.18	1.00	30 (96.8)				1.000 ^b^
	29	0.92±0.28	1.00	186 (91.6)	1.00±0.00	1.00	31 (100.0)				0.138^b^
	30	0.60±0.49	1.00	122 (60.1)	0.87±0.34	1.00	27 (87.1)				0.007*
	Domain score (%)	67.8±20.2	71.42		84.3±10.5	85.71		109.14/172.26	-4.876	0.000*	
	Total (%)	63.6±17.4	66.67		80.9±8.7	83.33		107.67/181.84	-5.694	0.000*	

* Statistically significant at p<0.05, The Mann-Whitney U-test was conducted for continuous variables and the chi square was conducted for categorical variables.

^a^ Chi-square test

^b^ Fisher’s exact test was used as the number of cells with expected count less that 5 is more than 20% of the total number of cells

### Factors associated with knowledge

Knowledge was higher in patients who completed their high school education, and patients who conducted fall prevention activities (R^2^ = 0.208, F = 3.949, df = 18, p<0.001). These two factors explained 27.9% of the variances.

### Comparison of the Osteoporosis Prevention And Awareness Tool (OPAAT) with other validated instruments

The OPAAT had a similar Flesch reading ease as the MOKT. The Cronbach’s α if the OPAAT ranged from 0.27–0.75 which was similar to the MOKT, Osteoporosis and you, OKAT and FOOQ which ranged from 0.60–0.82. This shows that the psychometric properties of the OPAAT were similar to that of other validated instruments for measuring patients’ knowledge (Table 11).

**Table 11:** Comparison of psychometric properties of the Osteoporosis Prevention And Awareness Tool (OPAAT).

	OPAAT	MOKT	Osteoporosis and You	OKAT	FOOQ	OKQ	OPQ
Age (years)	50–79	49–84	65–90	25–44	-	≥ 60	≥ 50
Number of subjects	203	88	871	467	256	188	50
Number of items with low difficulty level (%)	4(13.3)	19 (47.5)	6 (60)	3(15)	-	-	(44)
Flesch reading ease	59.2	57	-	45	81–90	-	74.3
Cronbach’s α or Kuder Richardson (KR)	0.27–0.75	0.82	0.60	0.70	0.76	0.80 (KR)	0.84 (KR)
Mean score (%)	63.6	69.0	37.7	44.0	-	57.4	-

OPAAT: Osteoporosis Prevention And Awareness Tool; MOKT: Malaysian Osteoporosis Knowledge Test [11], Osteoporosis and You [9]; OKAT: Osteoporosis Knowledge Assessment Tool [6]; FOOQ: facts on Osteoporosis Quiz [3,4]; OKQ: Osteoporosis Knowledge Questionnaire [10]; OPQ: Osteoporosis Questionnaire [7]

## Discussion

The OPAAT performed satisfactorily in its psychometric properties and was able to discriminate between knowledge level of patients and pharmacists. This indicates that the English version of OPAAT is suitable to assess knowledge of postmenopausal women about osteoporosis prevention in Malaysia.

EFA confirmed that there were three domains (osteoporosis in general, consequences of untreated osteoporosis and osteoporosis prevention) in the OPAAT to assess patient’s knowledge on osteoporosis and its prevention. This provides support for the construct validity of our tool. To the best of our knowledge no other osteoporosis knowledge assessment tool has validated the construct of their tool via this method.

Flesch reading ease was at 59.2. This indicates the OPAAT can be understood by patients who have completed primary education. Since all of our participants have completed primary education, they were able to complete the OPAAT without any problems. The mean ± SD accuracy rate was 0.60±0.22 (range:0.26–0.94). Out of the 30 items, four items were considered difficult (accuracy rates <0.3) and five considered easy (accuracy rates >0.7). The optimum difficulty level would be 0.5. This indicates that the OPAAT was moderately easy for the participants to answer.

The construct of the tool was considered to be multi-dimensional and an overall Cronbach’s α was unsuitable. We then analyzed the Cronbach’s α by domain. All domains demonstrated good and acceptable internal reliability except the domain on the ‘consequences of untreated osteoporosis’ with a Cronbach α value of 0.286. This could be because there were only 5 items in this domain, and knowing the correct answer for one item may not necessarily mean that they knew the correct answer for the next item. However, increasing the number of items within the domain would have made the questionnaire too lengthy reducing the likelihood of completion. Corrected item-total correlations showed that all items measured the same main component which is satisfaction except items 13/30(43.3%). However all items were retained as removing any of the items did not improve the overall Cronbach’s α significantly.

All 30 items performed satisfactorily at test-retest. Kappa measurement of agreement showed that 29/30 items (96.7%) were in very good agreement, and 1/30 items (3.3%) was in good agreement. As for the domains all domains performed satisfactorily except for the domain on “consequences of untreated osteoporosis.” Patients may have forgotten the answer they selected at test (as they might have been guessing) as opposed to knowing the right answer. This led to a significant difference in this domain score as it had a small number of items. Although this limits how well this domain can measure the knowledge on the consequences of untreated osteoporosis, the guessing of answer reflects actual practice. Nonetheless, there was no significant difference in the overall scores. This indicates the OPAAT has achieved stable reliability. The domains and items had a high Spearman’s rho correlation coefficient ranging from 0.760–0.990. They were all significantly correlated at p<0.05. Therefore, all items were retained.

Although pharmacists were expected to have a higher score than patients for all items, there were three items (items no. 13, 17 and 23) where no significant difference was found. This may be because more than 80.0% of both patients and pharmacists correctly answered items no. 13 and 23, indicating that their knowledge level for these items were high. As for item no. 17 which was pertaining to calcium intake, less than 60.6% of patients and pharmacists answered this item correctly. This concurs with our previous qualitative findings that found that both patients and pharmacists lacked knowledge in this area. [28]. Nonetheless, the overall score and all domain scores of the OPAAT showed a significant difference between the patient and pharmacist group. This indicates that the OPAAT has achieved discriminative validity.

Previous studies have found that the knowledge of osteoporosis in adult women aged 21–90 years in Europe [43,44,45], Canada [9], United States [5,23], Middle East [46], and Australia [6] was low. Conversely, women and men aged 16–79 years in Norway were knowledgeable about osteoporosis [47]. In Asia, the knowledge of osteoporosis ranged from low to moderate for women aged 19–90 in Brunei [48], Singapore [49] and Malaysia [27,50,51]. However, another study in Malaysia found that the knowledge of osteoporosis was moderate in women aged 49–84 [11]. In our study, patients’ overall knowledge score was 63.6±17.4, which indicate that their knowledge level was moderate. Our results were similar to a previous study conducted in Malaysia which assessed knowledge on osteoporosis and its prevention [11]. This may be because both studies were conducted in the same setting. In addition, participants in both studies were mainly health seeking urban patients.

However, we would like to highlight that the cohort of patients used in the Lai et al study was on patients who had osteoporosis, whilst our cohort were patients who were did not have osteoporosis. This shows that there was no difference in knowledge in patients with or without osteoporosis. Another tool, the Osteoporosis Knowledge Questionnaire (OKQ) assessed knowledge on osteoporosis risk factors, diagnosis, prevention and treatment in female population aged 60 and above scored 57.4% [10]. The OKQ score was similar to the OPAAT as they assessed non-osteoporotic postmenopausal population of a similar age group. Additionally, we would like to highlight the lack of knowledge on osteoporosis occurs in women who have not experienced a fracture, as well as those who had a previous fracture [52]. The different tools used to assess knowledge and the different cohorts in which the tool was administered to [11,27,50,51] made comparison between studies difficult. In addition, most studies did not report the use of validated tools to assess knowledge levels [23,24,25,43,44,45,47,48,50,51]

Patients’ knowledge was lowest on the domain on the ‘consequences of untreated osteoporosis.’ This concurs with findings from our qualitative research which indicates that there is a need to educate patients in this area [28]. Correspondingly, Osteoporosis and You noted a deficit in knowledge in the area of consequences of untreated osteoporosis [9]. These tools were developed mainly to assess the knowledge of domains of osteoporosis in general and treatment, the OPAAT was developed specifically to evaluate osteoporosis prevention.

In our study, factors with a positive correlation to the knowledge score includes patients with a secondary or higher education level, and patients who conducted fall prevention activities. Similarly, a Greek and Turkish study noted an association with knowledge and level of education [43,44,51]. Additionally, Khan et al’s findings concurred with our study as they noted a significant association between knowledge and ethnicity [51]. Conversely, Ailinger et al stated neither education level, age nor the menopause status increase osteoporosis knowledge [5]. Patients who conduct fall preventive measure had more knowledge of osteoporosis. This further justifies the importance of a higher knowledge level about osteoporosis prevention to ensure its implementation.

One of the limitations of our study was that convergent validity could not be performed. This was because during the period of our study, no such tool exists. The participants that we recruited also did not represent the ethnic distribution of Malaysia, but it represented the patients who sought treatment in our study site. Nonetheless, a large proportion of our patients had a monthly household income above $1553 (39.9%) which was representative of the married Malaysian household population income [53]. Seventy six percent of our participants were married. [53]. This shows that our participants income were representative of the Malaysian population.

Another limitation of our study was that we used mixed methods of administration. At baseline, majority of participants answered the OPPAT themselves, whilst a minority (2.5%) required assistance. At retest, the OPAAT was administered over the telephone as we wanted to optimize response rates. There is a possibility that participants may answer the items differently due to the mixed modes of administration [54]. However, this effect would be applicable to all participants, hence its effects on the validation process would be negated.

## Conclusion

The English version of the OPAAT was found to be a reliable and valid instrument for assessing patient knowledge on osteoporosis and its prevention in Malaysia. OPAAT can subsequently be used to evaluate the effectiveness of the education efforts provided. Future studies, using Bahasa Malaysia and Mandarin versions of the questionnaire are required to assess patient knowledge for Malaysians that are not fluent in English.

## Supporting Information

S1 Table Sample of the Osteoporosis Prevention and Awareness tool. (DOCX)
