# Supplementary material for: The Development and Validation of the Osteoporosis Prevention and Awareness Tool (OPAAT) in Malaysia
Source: PLoS One. 2015 May 4;10(5):e0124553. doi: 10.1371/journal.pone.0124553 (PMC4418569; doi:10.1371/journal.pone.0124553)
Supplement: S1 Table — (DOCX) [file pone.0124553.s001.docx]

**S1 Table: Sample of the Osteoporosis Prevention and Awareness TOOL (OPAAT)**

| Osteoporosis Prevention And Awareness Tool (OPAAT) | Serial no |  |  |  |
| --- | --- | --- | --- | --- |

Total score___________

Please tick at the appropriate box:

#### A. What can you tell me about osteoporosis?

|  | True_1_ | False_2_ | Don’t know_3_ |
| --- | --- | --- | --- |
| 1. Makes bones weaker, more brittle and more likely to break (fracture) |  |  |  |
| 1. Everybody will get osteoporosis as it is part of aging |  |  |  |
| 1. Osteoporosis occurs because bone is removed faster than it is formed |  |  |  |
| 1. Osteoporosis and osteoarthritis are different names we can use to describe the same disease |  |  |  |
| 1. Osteoporosis usually has no symptoms |  |  |  |
| 1. Postmenopausal women are not at risk for osteoporosis |  |  |  |
| 1. Osteoporosis is an untreatable disease. |  |  |  |
| 1. A bone mineral density test is used to diagnose osteoporosis |  |  |  |
| 1. I do not need a bone mineral density test unless I fracture my bones. |  |  |  |
| 1. A bone mineral density test is high in radiation |  |  |  |
| 1. A bone mineral density test should be performed monthly to monitor bone loss |  |  |  |

B. What will happen if your osteoporosis is left untreated?

|  | True_1_ | False_2_ | Don’t know_3_ |
| --- | --- | --- | --- |
| 1. Results in back pain |  |  |  |
| 1. Loss of height or hunchback |  |  |  |
| 1. Loss of mobility (unable to move around myself) |  |  |  |
| 1. Results in tooth loss |  |  |  |
| 1. Results in joint pain or swelling of fingers |  |  |  |

C. What can you tell me about osteoporosis prevention?

|  | True_1_ | False_2_ | Don’t know_3_ |
| --- | --- | --- | --- |
| 1. The recommended daily intake for calcium in women above 50 years of age is 1000mg |  |  |  |
| 1. It is too late to increase calcium intake after the age 50 |  |  |  |
| 1. Glucosamine can help prevent osteoporosis |  |  |  |
| 1. Calcium supplements can help prevent osteoporosis |  |  |  |
| 1. The regular dose of calcium supplements can cause kidney stones. |  |  |  |
| 1. Foods such as milk, tofu, anchovies (*ikan bilis*), yellow dhal and spinach are rich in calcium |  |  |  |
| 1. You can obtain your recommended daily intake of vitamin D via exposing your skin to sunlight for about 15 minutes a day |  |  |  |
| 1. Increasing coffee and tea intake can help in osteoporosis prevention |  |  |  |
| 1. Weight bearing exercise (such as brisk walking and line dancing) can decrease bone loss. |  |  |  |
| 1. Exercise will wear out bones |  |  |  |
| 1. Certain medications (such as sleeping tablets or high blood pressure medications) may reduce the risk of falling |  |  |  |
| 1. To prevent falls, comfortable shoes with a good grip should be used. |  |  |  |
| 1. Poor vision may lead to falls |  |  |  |
| 1. Being under weight helps prevent osteoporosis |  |  |  |
